# Rapid Redistribution of Synaptic PSD-95 in the Neocortex In Vivo

**DOI:** 10.1371/journal.pbio.0040370

**Published:** 2006-11-07

**Authors:** Noah W Gray, Robby M Weimer, Ingrid Bureau, Karel Svoboda

**Affiliations:** Howard Hughes Medical Institute, Cold Spring Harbor Laboratory, Cold Spring Harbor, New York, United States of America; Salk Institute for Biological Studies, United States of America

## Abstract

Most excitatory synapses terminate on dendritic spines. Spines vary in size, and their volumes are proportional to the area of the postsynaptic density (PSD) and synaptic strength. PSD-95 is an abundant multi-domain postsynaptic scaffolding protein that clusters glutamate receptors and organizes the associated signaling complexes. PSD-95 is thought to determine the size and strength of synapses. Although spines and their synapses can persist for months in vivo, PSD-95 and other PSD proteins have shorter half-lives in vitro, on the order of hours. To probe the mechanisms underlying synapse stability, we measured the dynamics of synaptic PSD-95 clusters in vivo. Using two-photon microscopy, we imaged PSD-95 tagged with GFP in layer 2/3 dendrites in the developing (postnatal day 10–21) barrel cortex. A subset of PSD-95 clusters was stable for days. Using two-photon photoactivation of PSD-95 tagged with photoactivatable GFP (paGFP), we measured the time over which PSD-95 molecules were retained in individual spines. Synaptic PSD-95 turned over rapidly (median retention times *τ*_r_ ~ 22–63 min from P10–P21) and exchanged with PSD-95 in neighboring spines by diffusion. PSDs therefore share a dynamic pool of PSD-95. Large PSDs in large spines captured more diffusing PSD-95 and also retained PSD-95 longer than small PSDs. Changes in the sizes of individual PSDs over days were associated with concomitant changes in PSD-95 retention times. Furthermore, retention times increased with developmental age (*τ*_r_ ~ 100 min at postnatal day 70) and decreased dramatically following sensory deprivation. Our data suggest that individual PSDs compete for PSD-95 and that the kinetic interactions between PSD molecules and PSDs are tuned to regulate PSD size.

## Abstract 2

Using two-photon microscopy and photoactivation of a fluorescently tagged synaptic protein (PSD-95), the authors demonstrated rapid turnover of these molecules in dendritic spines of the mouse sensory cortex in vivo.

## Introduction

Most excitatory synapses terminate on dendritic spines [1,2], tiny membrane protrusions that contain the postsynaptic density (PSD). In the rodent neocortex, spines begin to develop during the first postnatal week and increase in number during the first month of life [2–4], coincident with the development of synapses [5] and functional circuits [6,7]. Spine stability increases with developmental age [8–10]. In the adult brain, a subset of spines and their synapses can persist for months and maintain their size [11–14]. Spines vary in size, and their volumes are proportional to the area of the PSD [15] and synaptic strength [16–18]. The PSD is a proteinaceous complex composed of receptors, adhesion molecules, and signaling complexes [19–21]. In cultured neurons, PSD proteins exhibit turnover, degradation, and trafficking over hours [22–25]. How can synapses maintain their size and strength over months with unstable constituents?

To begin to address this question, we studied the dynamics of PSD-95 in single PSDs in vivo [26]. We focused on PSD-95 because it is a major organizer of the PSD. PSD-95 is part of a family of multi-domain PDZ-domain scaffolding proteins [21]. It is the most abundant PSD component [20,27,28] and has multiple binding partners in the synapse [21]. PSD-95 binds NMDA-Rs [29,30] and also interacts with AMPA-Rs via TARPs [31,32]. PSD-95 multimerizes [33] and appears very early at nascent synapses [34,35] where it clusters NMDA-Rs [30,36]. PSD-95 is thought to provide “slots” for AMPA-Rs, and PSD-95 levels at individual synapses could thus determine synaptic strength [37–40]. Despite its important structural role, biochemical measurements in dissociated cultures have suggested that PSD-95 and other associated PSD proteins have short half-lives [23,40–42].

Here we used optical methods to probe the turnover of PSD-95 in single PSDs in vivo. PSD-95 was bound to individual PSDs for approximately 30 min before diffusing into the dendrite and to other synapses. The synaptic retention time was developmentally regulated and experience-dependent. We further analyzed the trafficking of PSD-95 at individual synapses and found that the kinetic parameters are intricately tuned so that larger spines hold onto PSD-95 for longer and are also more efficient at capturing diffusing PSD-95. Therefore, the kinetic interactions between PSD-95 and the PSD may help to determine PSD size.

## Results

### Stable PSDs in the Developing Neocortex In Vivo

**Figure 1:**
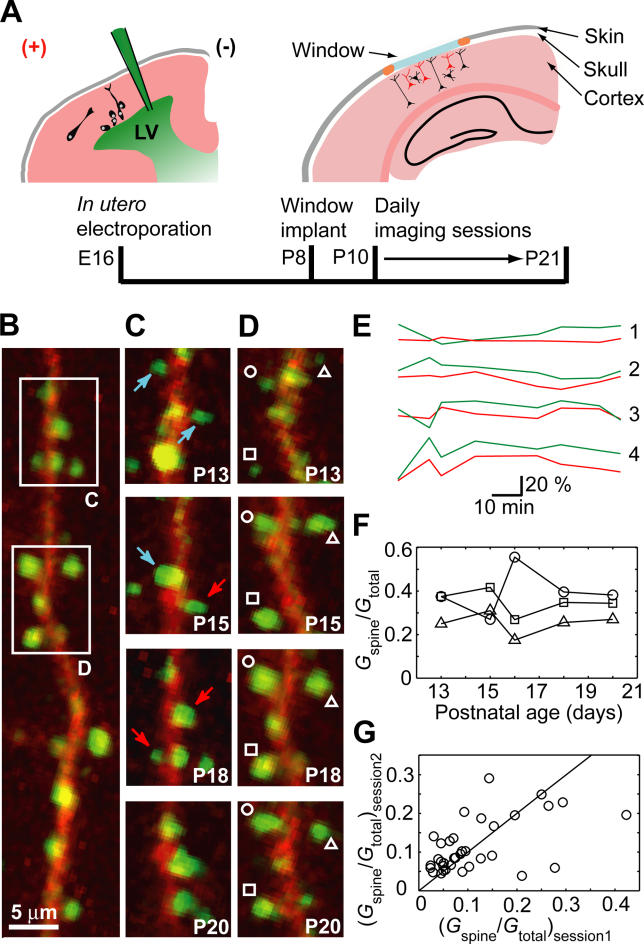
A Subpopulation of PSD-95 Clusters Is Stable over Several Days (A) Transfection of L2/3 pyramidal neurons by in utero electroporation. At E16, DNA was injected into the lateral ventricle (LV) and an electrical current was applied. At P8, imaging windows were implanted above the barrel field. High-resolution chronic imaging was performed from P10 to P21. (B) Dendritic segment transfected with PSD-95-GFP (green) and mCherry (red). (C) Time-lapse images showing turnover of PSD-95 clusters (box C, from [B]). Red arrows indicate gains; blue arrows indicate losses. (D) Time-lapse images showing stable PSD-95 clusters (box D, from [B]). (E) Fractional changes in PSD-95-GFP (green) and mCherry (red) fluorescence from four representative spines. Lines were offset for clarity. (F) Relative brightness of three PSDs (from [D]) over days. (*G_total_* = fluorescence summed over all spines). (G) Brightness of individual PSDs is correlated across two imaging sessions 1 d apart (*n* = 3 animals, 51 spines).

To probe the dynamics of PSD-95 clusters, we transfected layer (L)2/3 pyramidal neurons with the red fluorescent protein mCherry [43] and PSD-95-GFP [44] using in utero electroporation [45,46] (Figure 1A). An imaging window was implanted above the somatosensory cortex, allowing for daily high-resolution in vivo imaging [47]. It has been shown that the overexpression of PSD-95 can enhance synaptic strength [38,39] and spine size [48]. However, analysis of spine morphology and synaptic strength in brain slices harvested from the experimental mice revealed that, under our conditions, the expression of PSD-95-GFP in vivo did not change synaptic strength or spine size (Figure S1). Using dual-laser two-photon laser scanning microscopy, we imaged the structure of dendrites and their spines (red mCherry fluorescence, *R*) and the distribution of PSD-95 (green GFP fluorescence, *G*) (Figure 1B–1D). Green fluorescence was concentrated in puncta, with negligible diffuse fluorescence [44,49]. Most PSD-95-GFP puncta were in the tips of dendritic spines.

During the second week of life, L2/3 circuitry undergoes rapid experience-dependent development [5–8]. Long-term imaging revealed that spines appear and disappear [8] from day to day, consistent with high rates of synapse formation and elimination (Figure 1C). The fraction of spines gained or lost decreased with developmental age (fractional daily turnover: postnatal day (P)10–P13, 0.48 ± 0.06, *n* = 5 animals, 335 spines; P14–P16, 0.39 ± 0.05, *n* = 5 animals, 523 spines; P17–P21, 0.33 ± 0.04, *n* = 4 animals, 257 spines). Despite this rapid turnover of dendritic spines, some developing spines and their PSDs exhibited considerable stability. The PSD-95-GFP signal intensity in individual spines fluctuated little over imaging sessions lasting 90 min ((*G* − 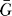 )/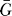 = 14 ± 11%, *n* = 9 spines; (*R* − 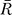 )/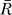 = 18 ± 14%, *n* = 9 spines) (Figure 1E). During these imaging sessions, we did not observe clear movements of PSD-95-GFP clusters in the dendrite, nor transitions of apparent shaft clusters to spine clusters and vice versa.

Spines that persisted over days typically had stable PSD-95-GFP clusters: over imaging sessions separated by one day, large (bright) PSD-95-GFP clusters were likely to remain large, whereas small (dim) PSD-95-GFP clusters remained small. Therefore the relative brightness of individual PSD-95-GFP puncta was highly correlated from day to day (Figure 1F and 1G) (*R* = 0.71, *p* < 0.01). These measurements show that a subpopulation of dendritic spines and their PSDs are remarkably stable as early as the second postnatal week of life.

### Rapid Redistribution of Synaptic PSD-95

**Figure 2:**
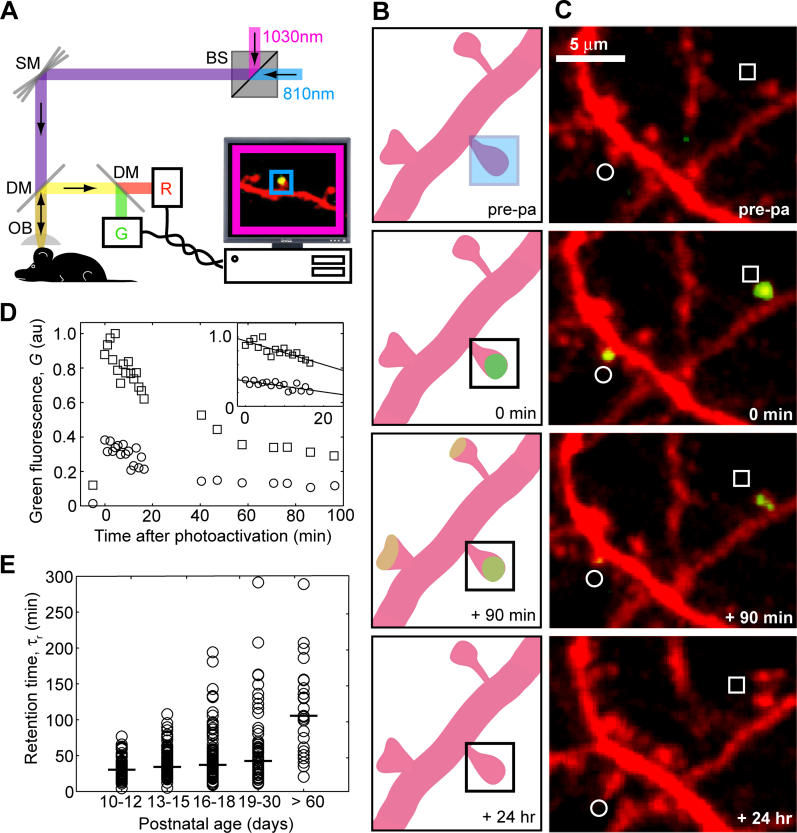
Rapid Turnover of PSD-95 in Individual PSDs (A) Schematic of the experimental setup. One laser (*λ* ~ 1,030 nm, magenta) was used to excite mCherry and photoactivated paGFP (paGFP^*^). A second laser (*λ* ~ 810 nm, blue) was used for photoactivation within a region of interest (blue box) within the field of view (magenta box). BS, beam-splitting cube; DM, dichroic mirror; OB, objective; R, G, photomultiplier tubes; SM, scan mirror. (B) Schematic of the experiment. Spines were selected for photoactivation (pre-pa, blue box), followed by time-lapse imaging. (C) Images collected before, and after (0 min, 90 min, and 24 h) photoactivation of two spines on different dendrites (age, P17). (D) Time course of PSD-95-paGFP* fluorescence (same as in [C]). The initial portion of the decay (inset) was used to extract the retention time (*τ*_r_) of PSD-95-paGFP^*^. (E) Retention times. Circles represent single spines and the horizontal bars indicate the medians (*τ*_median_). (P10–P12: *τ*_median_ = 30 min, range 4–77 min, *n* = 6 animals, 74 spines; P13–P15: *τ*_median_ = 34 min, range 5–108 min, *n* = 6 animals, 108 spines; P16–P18: *τ*_median_ = 37 min, range 9–194 min, *n* = 8 animals, 95 spines; P19–P30: *τ*_median_ = 43 min, range 11–291 min, *n* = 8 animals, 59 spines; >P60: *τ*_median_ = 106 min, range 20–289 min, *n* = 4 animals, 25 spines).

To explore the mechanisms that contribute to the maintenance of PSD-95 puncta, we tagged PSD-95 with photoactivatable GFP (paGFP) [50]. Under baseline conditions, paGFP showed negligible fluorescence (excitation wavelength *λ* ~ 1,030 nm) (Figure 2A–2C, pre-pa). Brief two-photon excitation at *λ* ~ 810 nm irreversibly converted the dark paGFP into a bright fluorophore (paGFP*) (Figure 2B and 2C, pre-pa vs. 0 min), revealing green puncta in photoactivated spines. Within photoactivated spines, PSD-95-paGFP* fluorescence decayed with an exponential time course over tens of minutes, followed by a long tail (Figure 2D). The exponential component reflects escape of PSD-95-paGFP* from individual spines; the long tail after the exponential decay is expected for trapped diffusion along the dendrite (proportional to *t^−^* ^½^; Equation S1, Protocol S1). One day after photoactivation, green fluorescence was undetectable (Figure 2B and 2C; 24 h).

The retention time (*τ*_r_), defined as the apparent time constant for the exponential component of the fluorescence decay (see Materials and Methods), measures the average time over which PSD-95 molecules are associated with individual spines. Although the initial phase of the fluorescence decay could be fit with a single exponential, multiple time constants, reflecting the complex and heterogeneous behavior of PSD-95 in single spines, likely shape the fluorescence decay. These measurements demonstrate that PSD-95 is retained by individual PSDs for 1 h or less, much shorter than the lifetime of dendritic spines and their PSDs and shorter than the half-life of PSD-95 (approximately 36 h) [23,41].

With increasing developmental age, PSD-95 became less dynamic. The retention time increased gradually (P10–P30; *R* = 0.50, *p* < 0.0002) (Figure 2E), resulting in approximately a 3-fold increase from early postnatal development (P10, *τ*_median_ = 22 min) to adulthood (>P60, *τ*_median_ = 105 min). The short retention times early during development could reflect the high plasticity potential of young cortical synapses during the barrel cortex critical period [7,8]. Note that at particular developmental ages, retention times were broadly distributed, with values spanning over one order of magnitude across spines (Figure 2E).

### The Retention Time Reflects Interactions of PSD-95 with the PSD

What mechanisms keep PSD-95-paGFP* in the spine and thus determine *τ*_r_? The retention time of PSD-95-paGFP* could reflect unbinding of PSD-95 from the PSD (with time constant *τ*_off_ = 1/*k*_off_). Alternatively, PSD-95 could be trapped in the spine head because of diffusional compartmentalization by the narrow spine neck (with time constant *τ*_esc_ = *V*_sp_ *ℜ*_n/_ *D*_o_ *,* where *V*_sp_ is the spine volume, *ℜ*_n_ the diffusional resistance of the spine neck, and *D*_o_ the free diffusion coefficient of PSD-95-GFP) [51,52]. To distinguish between these possibilities, we measured retention times for other proteins that are not known to be concentrated in the PSD.

**Figure 3:**
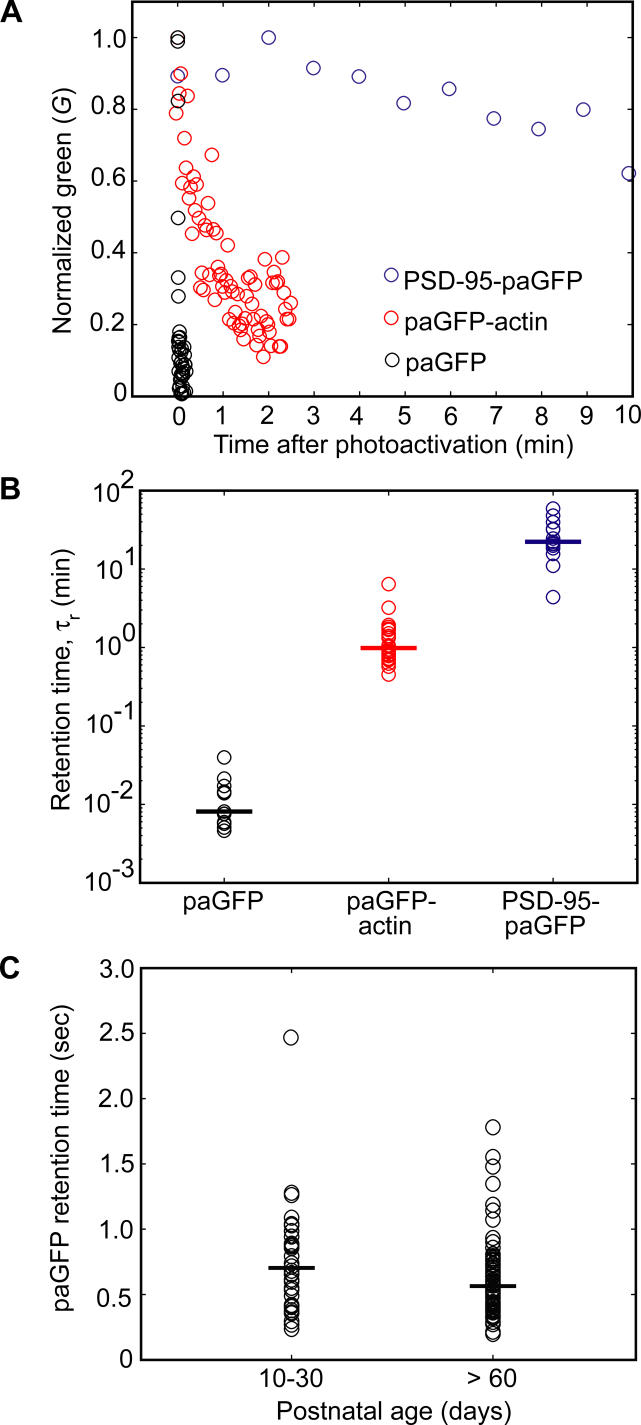
The Magnitude of the PSD-95 Retention Time Is Determined by Its Interactions with the PSD (A) Examples of the time course of paGFP* (black), paGFP*-actin (red), and PSD-95-paGFP* (blue) fluorescence after photoactivation in single spines (age, P10). (B) Retention times for paGFP*, paGFP*-actin, and PSD-95-paGFP* (age, P10) (circles indicate individual spines; bars indicate medians) (median *τ*_paGFP_ = 0.47 s, range, 0.28–1.28 s, *n* = 2 animals, 11 spines; median *τ*_paGFP-actin_ = 0.98 min, range, 0.45–6.42 min, *n* = 3 animals, 26 spines; median *τ*_PSD-95-paGFP_ = 22 min, range, 4–59 min, *n* = 2 animals, 15 spines). (C) Escape time for paGFP* from individual spines (circles) as a function of age. (P10–P30: *τ*_median_ = 0.72 s, range 0.28–2.38 s, *n* = 4 animals, 35 spines; > P60: *τ*_median_ = 0.58 s, range 0.24–1.78 s, *n* = 1 animal, 105 spines).

The retention time of cytoplasmic paGFP* (*τ*_paGFP_) is determined by spine geometry alone (Protocol S1), whereas the retention time of paGFP*-actin (*τ*_paGFP-actin_) in addition depends on the cycling of actin in dendritic spines [53,54]. Since the diffusion coefficient is only a weak function of molecular weight (~MW^1/3^) [55], paGFP*, paGFP*-actin, and PSD-95-paGFP* are expected to have similar (within a factor of 2) values for *τ*_esc_. The retention times for paGFP* were about 1,000 times shorter than for PSD-95-paGFP* (median *τ*_paGFP_ = 0.47 s) (Figure 3A and 3B) and was independent of developmental age (Figure 3C), but increased with spine volume (Figure S2) [52]. The retention time for paGFP*-actin was intermediate (median *τ*_paGFP-actin_ = 0.98 min) (Figure 3A and 3B) [56]. Therefore the magnitude of *τ*_r_ is set primarily by the interactions of PSD-95 with its binding partners in the PSD and only modulated by compartmentalization of diffusing PSD-95-paGFP* by spine necks (see Equation 1 in Discussion).

### Synapses Share a Common Pool of Diffusing PSD-95

**Figure 4:**
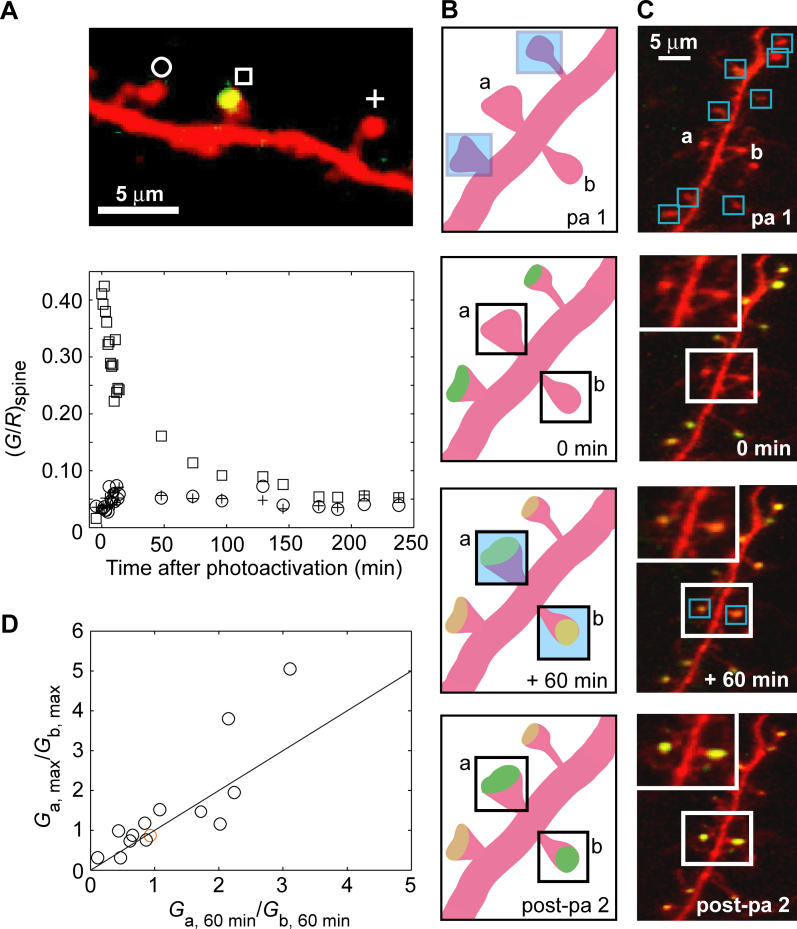
Individual PSDs Share a Common Pool of PSD-95 (A) Photoactivation of PSD-95-paGFP in a single spine (square) is followed by an increase in green fluorescence in neighboring spines (circle and cross). Top, image. Bottom, corresponding fluorescence time course. Green fluorescence was normalized to red fluorescence in the spine. (B) Measuring the relationship between PSD-95 capture and PSD size. All but two (pa 1, a and b) “probe” spines were photoactivated. The fluorescence intensity within the probe spines was quantified (*G*_a_ and *G*_b_) after 60 min. Subsequently the probe spines (a and b) were also photoactivated to estimate the sizes of their PSDs (post-pa 2, [*G*_a, max_ and *G*_b, max_]). (C) Time-lapse images corresponding to (B). Insets show the probe spines a and b*.* Note, the last image was acquired after the probe spines were fully photoactivated. (D) Larger PSDs capture more PSD-95. Comparisons were performed for pairs of spines, a and b. The PSD size ratio (*G*_a,max_/*G*_b,max_) was proportional to the ratio of capture (*G*_a,60min_/*G*_b,60min_) (orange circle corresponds to the spine pair in [C]).

We wondered if PSD-95 could exchange between PSDs in different spines. After photoactivating PSD-95-paGFP in a single spine, fluorescence appeared in neighboring spines as fluorescence decreased in the photoactivated spine (Figure 4A). Degradation of PSD-95 (half-life ~ 36 h) is negligible over the time course of single imaging sessions (90 min) [23,41]. The spread of PSD-95 was bidirectional and did not involve obvious transport particles. Furthermore, diffuse PSD-95 in the dendrite spread rapidly over short distances (Protocol S1). These observations argue that PSD-95 spreads from PSD to PSD by diffusion and individual PSDs share a common pool of diffusing PSD-95.

### Synapse-Specific Capture and Retention of PSD-95

What governs the diffusional exchange of PSD-95 between spines? After PSD-95 unbinds from the PSD, it diffuses rapidly (inter-synapse diffusion times ~ 50 ms, Protocol S1) along the dendritic shaft until it is captured by other PSDs. PSD-95 content is roughly proportional to PSD area [57,58]. Therefore large PSDs contain large PSD-95 clusters and are prominent sources of PSD-95, and the resulting concentration gradients along the dendrite will drive net PSD-95 flux from large PSDs towards small PSDs. Diffusion therefore tends to dissipate differences in the sizes of PSD-95 clusters. How can PSD size be maintained in the presence of diffusion? Stable sets of PSD-95 binding sites at individual PSDs could explain the stability of PSD-95 clusters. However, this explanation is likely not sufficient because PSD-95 binding partners are also unstable [23,59], with half-lives at synapses on the order of minutes to hours [60,61]. We therefore analyzed the parameters governing the exchange of PSD-95 between synapses to determine if synapses of different sizes have kinetic mechanisms to maintain the sizes of their PSD-95 clusters.

We examined the capture of PSD-95 by individual PSDs. Capture could be dominated by the spine neck: less-restrictive spine necks allow for more flux of PSD-95 between the dendrite and spine, facilitating the capture of diffusing PSD-95. PSD size should also play a role, since the number of binding sites for PSD-95 in the PSD is likely proportional to PSD size, larger PSDs could capture more PSD-95. We estimated the relationship between PSD-95 capture and PSD size. We photoactivated all but two spines on a small dendritic branch (spines a and b; Figure 4B and 4C). Spines a and b were chosen to be near each other and thus experienced similar concentrations of diffusing dendritic PSD-95-paGFP*. By measuring the green fluorescence accumulating in spines a and b (*G*_a_ and *G*_b_), we estimated how efficiently these spines captured PSD-95-paGFP* (Figure 4B and 4C, +60 min). We measured PSD-95-paGFP* fluorescence again after photoactivating spines a and b (*G*_a,max_ and *G*_b,max_). The green fluorescence immediately after photoactivation (*G*_a,max_ and *G*_b,max_) was used as a measure of the size of the PSD-95 cluster and hence as an estimate of PSD area [57,58]. The ratio of PSD-95-paGFP* capture (*G*_a,60min_/*G*_b,60min_) and PSD size (*G*_a,max_/*G*_b,max_) were highly correlated (*R* = 0.86, *p* < 0.0001) (Figure 4D). Therefore, PSDs captured diffusing PSD-95-paGFP* in proportion to their size.

**Figure 5:**
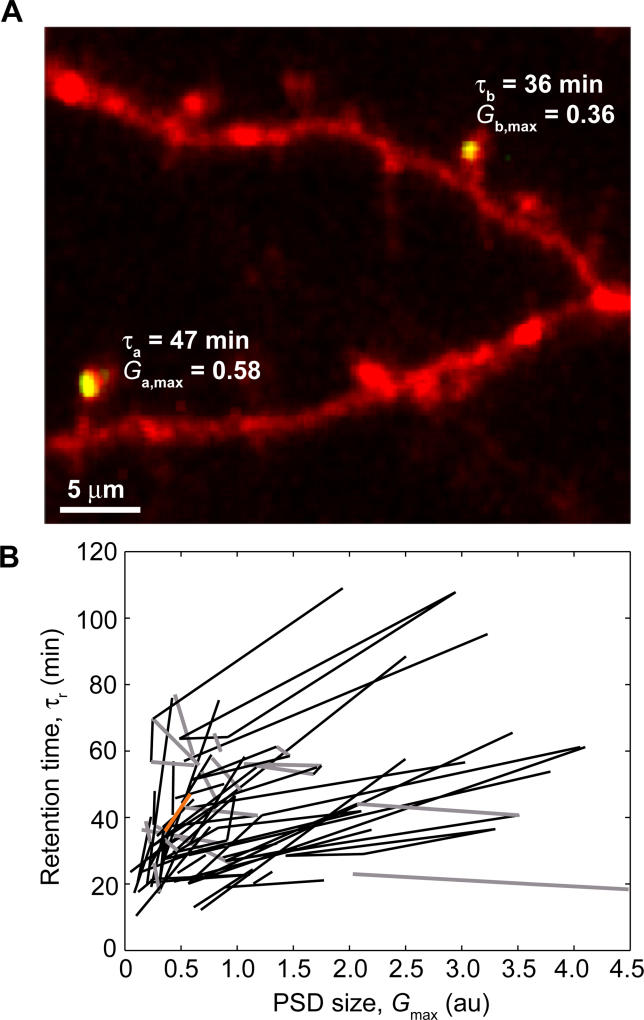
Larger Spines Retain PSD-95 Longer (A) Image of two photoactivated spines, a and b, on two branches of the same dendrite. *G_a_*_,max_ and *G_b_*_,max_ are the fluorescence intensities of PSD-95-paGFP^*^ immediately after photoactivation, a measure of PSD size. *τ*_a_ *,* and *τ*_b_ are the corresponding retention times. (B) Comparison of retention times and PSD sizes for pairs of spines. Each line corresponds to a pair of spines, a and b (black, positive slope, *n* = 64; gray, negative slope, *n* = 18, 6 animals; orange, example from [A]). Spines with larger PSDs have longer retention times.

We next studied the relationship between PSD size and the PSD's retention time for PSD-95-paGFP*. We compared retention times for pairs of spines (*τ*_a_ and *τ*_b_). The green fluorescence immediately after photoactivation (*G*_a,max_ and *G*_b,max_) was again used as a measure of PSD size. Both spines shared the same parent dendrite and thus also the same PSD-95-paGFP expression levels and imaging conditions. However, spines were chosen to lie on different dendritic branches to minimize mixing of PSD-95-paGFP* between spines (Figure 5A). In most cases the larger in a pair of PSDs retained PSD-95 longer (Figure 5B) (*p* < 0.02, Wilcoxon signed rank test). Therefore spines with larger PSDs capture more diffusing PSD-95 and retain PSD-95 for longer. At steady state, the size of individual PSD-95 clusters is expected to scale with the PSD's capture rate and retention time (Protocol S1).

### Changes in PSD Size over Time Predict Changes in Retention Time

**Figure 6:**
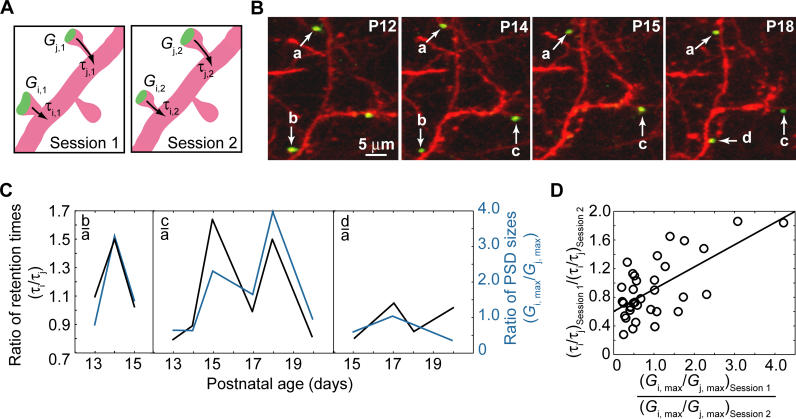
PSD Size and PSD-95 Retention Time Vary Together from Day to Day (A) Schematic of the experiment. Spines (i and j) on the same dendrite were photoactivated to measure PSD-95 fluorescence (*G*_i,j_) and the PSD-95 retention times (*τ*_i,j_). The ratios *G*_i_/*G*_j_ and *τ*_i_/*τ*_j_ were compared across imaging sessions. (B) Example of repeated imaging and photoactivation of three spines over 6 d. (C) Ratios of retention times (black) and ratios of PSD sizes (blue) for pairs of spines as a function of age (same experiment as [B]). (D) Changes in fluorescence retention time predict changes in PSD size between imaging sessions 1 d apart. The line is a least-squares fit to the data (*n* = 4 animals, 18 spine pairs, 35 2-d sequences).

Our measurements suggest that kinetic mechanisms tuned at the level of individual synapses could help maintain PSD size with dynamic PSD components. Larger PSDs hold onto PSD-95 for longer and capture PSD-95 more efficiently than smaller PSDs. Therefore, if *τ*_r_ partially determines the size of the PSD-95 cluster, then changes in retention time should co-vary with changes in the PSD-95 cluster. We measured PSD-95 clusters (*G*_max_ of photoactivated PSD-95-paGFP) and *τ*_r_ for groups of PSDs on the same dendritic tufts over several days (Figure 6A and 6B). For pairs of spines, the ratio of their retention times across imaging sessions (Figure 6C, black line) tracked the ratios of the PSD-95-paGFP* cluster brightness (Figure 6C, blue line). Over populations of spine pairs, changes in *τ*_r_ over time were highly correlated with changes in PSD-95 clusters (*R* = 0.66, *p* < 0.0001) (Figure 6D). These measurements show that the retention time for PSD-95 and the size of the PSD-95 cluster are tightly coupled at the level of single synapses.

### PSD-95 Stability Is Modulated by Sensory Experience

**Figure 7:**
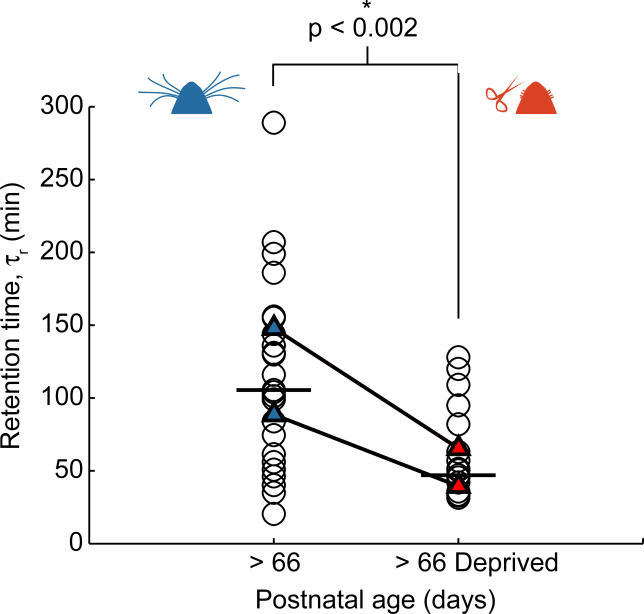
PSD-95 Retention Time Is Modulated by Sensory Experience Circles indicate individual spines (>P60 control data are repeated from Figure 2E). Horizontal bars indicate medians. Triangles indicate population averages for two animals in which retention times were measured before (blue) and after (red) whisker clipping.

Studies in cultured neurons suggest that the stability of PSD-95 at synapses can be modulated by synaptic activity [40–42,44]. We therefore tested if the turnover of synaptic PSD-95 is influenced by the level and pattern of network activity in vivo. The sensory input to the barrel cortex can be altered by trimming the mystacial whiskers that provide the main sensory input to the barrel cortex. Deprivation was initiated in adult mice (age > 60 d) and involved the clipping of the large whiskers on both sides of the snout. Retention times were measured in the same animals before and after whisker clipping, 1–30 d after clipping. Whisker clipping dramatically reduced the retention times of synaptic PSD-95 (median *τ*_r_ unclipped = 106 min; median *τ*_r_ clipped = 49 min; *p* < 0.002, Wilcoxon rank sum test) (Figure 7), demonstrating that the stability of synaptic PSD-95 is activity- and experience-dependent.

## Discussion

### Rapid Redistribution of Synaptic PSD-95 In Vivo

We used dual laser two-photon microscopy [56,62] and two-photon photoactivation of paGFP [52] to study the dynamics of PSD-95 in L2/3 dendrites in vivo. Our data show that PSD-95 is retained by individual PSDs for 1 h or less. This is approximately 50-fold shorter compared to the half-life of PSD-95 (~36 h) [23,41] and more than 1,000-fold shorter than the lifetime of persistent spines and their synapses (months) [9,11,13,14]. After PSD-95 unbinds from a PSD, it diffuses rapidly within the dendrite and binds to other PSDs (Figure 4A). The diffusion time of free PSD-95-paGFP between synapses (~50 ms, Protocol S1) is rapid compared to the retention time. We estimate that at most, 1% of PSD-95 is unbound at any given time. Therefore individual PSD-95 molecules effectively hop from spine to spine, visiting dozens of PSDs before being degraded. As a consequence, synapses share dendritic PSD-95.

Calculations and detailed simulations (unpublished data), constrained by the observed behavior of PSD-95, suggest that the concentration of PSD-95 binders exceeds the total concentration of PSD-95. Thus individual PSDs compete for a limiting pool of PSD-95; an increase in the size of the PSD-95 cluster in one spine occurs at the expense of neighboring PSDs (Figure S3).

PSD-95 levels may determine synaptic strength [38,39,63] and, together with other PSD molecules, set synapse size [48,64–66]. Redistribution of synaptic PSD-95 by diffusion could play a role in synaptic plasticity by rapidly reallocating synaptic resources to potentiated synapses within hours, before translational and transcriptional programs have time to respond [22,67]. Such redistribution of synaptic PSD-95 (or other PSD molecules, such as CaMKII [68], SAP97 [69], or Shank [60]) may underlie interactions between nearby synapses in the induction and maintenance of long-term potentiation [70].

### Kinetic Factors Contributing to Synapse Stability

In vitro studies suggest that most, if not all, proteins at an excitatory synapse exhibit half-lives similar to that of PSD-95 or shorter [60,61,71]. How, therefore, can stable synapses be maintained with unstable constituents? The synapse-specific regulation of PSD-95 capture and retention provides a case study on how synaptic stability could be achieved with highly dynamic synaptic proteins.

Large PSDs are large sources of diffusing PSD-95, and a net diffusional flux is expected to carry PSD-95 from large to small synapses. Without synapse-specific regulation of kinetic parameters, the interactions between PSD molecules and PSDs are identical in all spines; large PSDs would therefore lose material and size at the expense of small PSDs, until all PSDs have a similar size. Our data indicate that these parameters are tuned at the level of individual synapses to counteract these dissipative effects of diffusion. First, larger PSDs are more effective in capturing diffusing PSD-95 (Figure 4D). Second, larger PSDs retain PSD-95 for longer than smaller PSDs (Figure 5B), which helps maintain differences in the sizes of PSD-95 clusters. Interactions between PSD-95 molecules in the PSD are also likely required to maintain stable PSD-95 clusters [72]. Similar mechanisms may apply to other PSD proteins to set the PSD content for these proteins.

What could be the mechanisms underlying synapse-specific retention times? Although the interactions of PSD-95 with its binding partners in the PSD set the magnitude of *τ*_r_ (Figure 3), compartmentalization of PSD-95 by the spine can still influence the value of *τ*_r_. *τ*_esc_ (*= V*_sp_ *ℜ*_n_/*D*_o_) measures the compartmentalization by the spine neck [51,52]. After unbinding from the PSD, PSD-95 is trapped in the spine head for time *τ*_esc_ before escaping into the dendrite through the spine neck [51]. Trapping in the spine (for time *τ*_esc_) drives rebinding of PSD-95 to the PSD (with time constant *τ*_on_) before escape into the dendritic shaft. The retention time can be estimated as (Protocol S1):

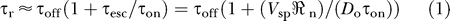
Therefore the retention time depends on *τ*_esc_ and, therefore, on spine geometry. Since *τ*_esc_ is expected to be on the same order as *τ*_on_ (Protocol S1), spine geometry can modulate the retention time (see also simulation, Figure S4); the retention time is expected to increase with spine volume (Figure S2) and spine neck resistance.

Spine geometry could explain the dependence of *τ*_r_ on PSD size. According to Equation 1, retention times are longer for spines with larger spine volumes (*V*_sp_). Since PSD size is proportional to spine volumes [73,74] (Figure S5), retention times are also expected to be longer for larger PSDs based on spine geometry alone.

Differences in retention times between spines could in addition be tuned by the biochemical time constants *τ*_on_ [72] and *τ*_off_ (Equation 1). Several mechanisms could contribute to the regulation of *τ*_on_ and *τ*_off_. (1) Larger PSDs could contain different PSD-95 binding partners and thus interact more strongly with PSD-95 compared to smaller PSDs; (2) Individual PSD-95 molecules simultaneously interact with multiple binding partners. It is possible that the full complement of binding partners is only available at the center of the PSD, whereas only a subset of binding partners may be available at the edge, implying weaker binding. Since the ratio of PSD area over PSD circumference increases with PSD size, large PSDs could bind PSD-95 more strongly; (3) PSD-95 association with the PSD is controlled by activity-dependent post-translational modifications, and these could be regulated differently at different synapses [33,41]. Additional experiments will be required to explore these possibilities directly.

### Developmental and Experience-Dependent Regulation of PSD-95 Stability

PSD-95 retention times increased steadily with developmental age (Figure 2E). It is unlikely that geometric factors relating to spine compartmentalization influence this increase since there was no change in *τ*_esc_ with age (Figure 3C). However, several prominent structural molecules that bind to PSD-95 directly or indirectly, including GKAP, Shank, and CaMKII, are developmentally regulated [75–78]. Developmentally regulated protein–protein interactions in the PSD therefore could underlie the developmental increase in retention times (through changes in *τ*_on_ or *τ*_off_ , Equation 1).

Whisker clipping reduces the synchronous activity in the neocortex [79] and in our experiments caused a reduction in PSD-95 retention times (Figure 7). These data show that PSD-95 stability at synapses is activity- and experience-dependent. Similarly, a number of dendritic proteins show activity-dependent trafficking and stability in cultured systems [54,60,61]. In our whisker-clipping protocol, the entire barrel cortex is deprived and the majority of synapses experience a reduction in activity, which is presumably the cause of the dramatic drop in the average retention time. In previous studies using a checkerboard pattern of whisker deprivation, subsets of synapses within the barrel cortex were stabilized whereas others were lost [14]. In more natural situations, novel experiences would likely increase activity at some synapses and decrease activity at others. The rapid redistribution of PSD-95, and other PSD molecules, could cause rapid shifts in synapse size and strength towards the more active inputs.

## Materials and Methods

### DNA constructs.

The expression vector for in vivo expression of synaptic proteins was a modified pCAGGS vector [80]. mCherry [43], PSD-95 tagged with monomeric EGFP (mEGFP) [44,81], or paGFP [50] was inserted within the coding region of pCAGGS, including a Kozak consensus sequence. The 3′ untranslated region contained a promoter-independent enhancer element (woodchuck hepatitis virus posttranscriptional regulatory element) [82] and the bovine growth hormone polyadenylation site. In some experiments, we also used plasmids expressing paGFP by itself or paGFP-actin based on chick beta-actin [83] from the same expression vector. All DNA was purified and concentrated using Qiagen plasmid preparation kits (Valencia, California, United States) and dissolved in 10 mM Tris–HCl (pH 8.0).

### In utero electroporation.

All experimental protocols were conducted according to the National Institutes of Health guidelines for animal research and were approved by the Institutional Animal Care and Use Committee at Cold Spring Harbor Laboratory. L2/3 progenitor cells were transfected via in utero electroporation [45,46]. E16 timed-pregnant C57BL/6J mice (Charles River, Wilmington, Massachusetts, United States) were deeply anesthetized using an isoflurane–oxygen mixture (1% vol isoflurane/vol O_2_) delivered by an anesthesia regulator (SurgiVet, Waukesha, Wisconsin, United States). The uterine horns were exposed and approximately 1 μl of DNA solution (containing ~2 μg/μl of plasmid expressing green protein, a molar equivalent of mCherry plasmid, and Fast Green [Sigma, St. Louis, Missouri, United States]) was pressure injected (General Valve Picospritzer, Fairfield, New Jersey, United States) through a pulled-glass capillary tube (Warner Instruments, Hamden, Connecticut, United States) into the right lateral ventricle of each embryo. The head of each embryo was placed between custom-made tweezer electrodes, with the positive plate contacting the right side of the head. Electroporation was achieved with five square pulses (duration = 50 ms, frequency = 1 Hz, 40V). Co-transfection efficiencies were 60%–70%.

### Surgery.

Imaging windows were installed above the somatosensory cortex at P8 [47] or after P60 [9,12]. Mice were deeply anesthetized with an isoflurane-oxygen mixture. A craniotomy (diameter ~ 3 mm) was opened above the right somatosensory cortex (0.5/1.5 mm posterior from bregma and 3.0/3.5 mm lateral from the midline for pups/adults, respectively), leaving the dura intact. The dura was covered with 1% agarose (Type-IIIA, Sigma) that was dissolved in HEPES-buffered artificial cerebrospinal fluid and covered with a 5-mm custom-made cover glass (No. 1) that was sealed into place with dental acrylic. The animals were also given a 20-μl injection of 4% dexamethasone (Phoenix Scientific, St. Joseph, Missouri, United States of America). After a 1-h recovery period, adults were replaced into the cage, and pups were housed with littermates and a surrogate mother. In the younger animals, mice with (*n* = 6 animals) and without (*n* = 9 animals) imaging windows gained weight at the same rate from P10 to P21 (*p* > 0.84, Wilcoxon rank sum test). For a subset of animals, the position of the imaging window was mapped relative to L4 barrels using standard histological methods.

### Imaging.

For younger animals, daily imaging began at P10. Windows in adult animals were allowed a week to stabilize before imaging sessions began. For deprivation experiments, whiskers were clipped every other day following the last non-deprived imaging session. Animals were anesthetized with an isoflurane-oxygen mixture and mounted to the microscope using a head post. High-resolution images were collected via a custom-made two-laser two-photon laser scanning microscope (2PLSM) [56,62]. The light source for imaging mCherry/paGFP* was a solid-state Ytterbium laser (*λ* ~ 1,030 nm; ~110 mW in the objective back-focal plane) (t-Pulse; Amplitude Systemes, Pessac, France); for imaging GFP we used a Ti:sapphire laser (*λ* ~ 910 nm; 25 mW) (MaiTai; Spectra Physics, Fremont, California, United States); for photoactivation of paGFP we utilized the Ti:sapphire laser (*λ* ~ 810 nm; 125 mW, using 2–3 frames, 3 μm in diameter, centered on spines). The laser beams were independently controlled using Pockels cells (350–80 LA; Conoptics, Danbury, Connecticut, United States) and combined using a polarizing beam splitting cube. One to five spines were photoactivated before image collection. Image acquisition and Pockels cell control was with ScanImage [84]. Red and green fluorescence photons were separated using a 565-nm dichroic mirror (Chroma Technology, Brattleboro, Vermont, United States) and bandpass filters (510/40; 635/90; Chroma Technology). Signals were collected using photomultiplier tubes (3896; Hamamatsu, Hamamatsu City, Japan). The objective lens (40, 0.8 NA) and trinoc were from Olympus (Tokyo, Japan).

We used the vasculature and the pattern of dendritic branching to identify regions of interest from day to day. Imaging sessions consisted of a series of image stacks over 90 min, with time-lapse intervals ranging from 1–20 min. Image stacks consisted of individual sections (256 × 256 pixels; pixel size, 0.16 μm) separated axially by 0.5 μm. For measurements of cytosolic paGFP and paGFP-actin, a series of single sections were collected 128 ms apart (64 × 64 pixels). After an imaging session, mice were allowed to recover for approximately 30 min on a warming blanket before housing with littermates and a surrogate mother; adult animals were returned to their cages.

### Data analysis.

Fluorescence intensities were quantified using custom software (MatLab [http://www.mathworks.com/products/matlab/]). For each spine, mean intensities (ROIs, ~1 μm) were further averaged over three sections (the brightest section plus one above and below). Unless otherwise stated, green fluorescence *(G)* was normalized by the red (mCherry) fluorescence summed over the whole dendrite (*R*_sum_), to correct for possible fluctuations in the imaging conditions. To estimate the size of the PSD-95 clusters in PSDs containing PSD-95-paGFP, we measured the green fluorescence approximately 1–2 min after saturating photoactivation and background subtraction.

To extract the retention time, *τ*_r_, *G*/*R*_sum_ was fit with an exponential function and an offset, *G*/*R*_sum_ = ae^−t/b^ + c. *τ*_r_ was derived over the interval 0 − *b* using a second round of exponential fitting as e^−t/*τr*^. *τ*_r_ derived in this way primarily measures retention by the PSD and spine rather than diffusional relaxation of PSD-95 in the dendrite (this was verified using simulations; see Protocol S1). *τ*_r_ is the average residence time of PSD-95 in a spine. It is likely that the underlying true decay is multi-exponential, reflecting heterogeneous interactions of PSD-95 with its binding partners. For experiments with paGFP-actin (Figure 3) *τ*_paGFP-actin_ was the slower of two time constants, representing actin recycling rather than diffusion of free actin [54]. *τ*_paGFP_ was calculated using a single exponential function [51].

Capture rates were estimated by comparing the PSD-95-paGFP* fluorescence for pairs of spines that were next to a group of spines that were photoactivated 60 min earlier (Figure 4). Over this time, fluorescence in individual spines may have been influenced by retention, in addition to capture. However, the fluorescence that accumulated by transfer was only weakly dependent on time 20–60 min after photo-activation (Figure 4A), and the fluorescence signal at 60 min is therefore an accurate measure of the rate of capture.

Spine lifetimes, densities, and lengths were measured using custom software [9]. Spine densities in cells expressing PSD-95-XFP and mCherry (0.30 ± 0.06 spines/μm dendrite) were indistinguishable from cells expressing only mCherry (0.28 ± 0.09 spine/μm dendrite; *p* > 0.31, Wilcoxon rank sum test). Similarly, spine lengths were indistinguishable (PSD-95-XFP/mCherry, 1.51 ± 0.68 μm; mCherry, 1.35 ± 0.47 μm; *p* > 0.26, Wilcoxon rank sum test) (Figure S1G and S1H). The fractional daily turnover was calculated as (*N*_gained_ *+ N*_lost_)/*N*_total_, where *N*_total_ represents the combined number of spines on the two days being compared. Note that mCherry fluorescence was relatively dim and further decreased with developmental age; it is possible that we may have missed the smallest spines in our analysis.

## Supporting Information

Figure S1 Synaptic Circuits and Physiological Properties Are Not Affected by the Expression of PSD-95 (A) Comparison of resting membrane potentials of transfected and non-transfected cells in L2/3 in P15 brain slices.
(B) Comparison of membrane capacitance.
(C) Comparison of input resistance.
(D) Examples of laser scanning photostimulation input maps from two L2/3 cells (left, transfected; right, non-transfected). The color map indicates the strength of synaptic input from particular locations in the brain slice (red dot, soma position; dashed lines, barrels; black pixels, direct responses from the patched cell).
(E) Paired comparisons of the average synaptic input to transfected and non-transfected cells in the same slice. Red dot indicates the mean (*n =* 9 pairs, four animals).
(F) Paired comparisons of the average L4 input from transfected and non-transfected cells, input representing only non-transfected cells. Red dot indicates the mean of all pairs (*n =* 9 pairs, four animals).
(G) Comparison of spine density for PSD-95-XFP/mCherry expressing cells (*n* = 4 animals, 284 spines, 940 μm total dendritic length) and mCherry expressing cells (*n* = 3 animals, 512 spines, 1,811 μm total dendritic length). Data collected at P15.
(H) Comparison of spine lengths for PSD-95-XFP/mCherry expressing cells (*n* = 4 animals, 284 spines) and mCherry expressing cells (*n* = 3 animals, 512 spines). Data collected at P15.
Error bars for (A–C), (G), and (H) represent standard deviation. Error bars in (E) and (F) represent standard error of the mean for each cell in the pair.
(5.4 MB TIF)

Figure S2 Escape Time (*τ*_esc_) Increases with Spine Volume *n* = 1 animal and *n* = 6 spines at P55, 4 spines at P56, and 6 spines at P57.
(1.9 MB TIF)

Figure S3 Simulation of Synaptic Competition for PSD-95 Time course of PSD-95 fluorescence; the PSD in the spine highlighted in blue has an affinity for PSD-95 2× greater than its neighbors. Inset: two-dimensional geometry. Reaction parameters: *k*_on_ = 10/μM . sec; *D_o_* = 10 μm/sec^2^. Initial concentrations (in μM): bound PSD-95 = 50, free binding sites = 5; ubiquitous unbound dark PSD-95 = 8.3 . 10^−4^. PSD-95 affinity (*k*_off_) = 1/2,400 sec (blue) or 1/1,200 sec (remaining spines).
(2 MB TIF)

Figure S4 Simulations of PSD-95 Redistribution (A) Schematic of dendrite (bottom) and a blow-up of a dendritic spine (top).
(B) State diagram for PSD-95.
(C and D) Monte Carlo simulation of photoactivation experiments. The simulations are based on the state diagram of Figure S4B. Each simulation represents 1,000 PSD-95-paGFP molecules.
(C) Time course of fluorescence in the photoactivated spines (solid lines) and in their direct neighbors (dotted lines). Parameters (in seconds): *τ*_on_ = 1; *τ*_off _= 1,000; *τ*_diff_ = 0.1; *τ*_in_ = 5 *τ*_esc_. *τ*_esc_ was 0.1 (green), 0.5 (red), or 2 (blue). The estimated retention times were 1,303, 1,605, and 3,027 s.
(D) Retention times as a function of *τ*_off _. Parameters (in seconds): *τ*_on_ = 1; *τ*_esc_ = 2; *τ*_diff_ = 0.1; *τ*_in_ = 5 *τ*_esc_. For each parameter value the simulation was repeated twice.
(E and F) Compartmental modeling using the Virtual Cell Modeling and Simulation Framework (University of Connecticut Health Center).
(E) Inset: two-dimensional geometry. The diameter of each spine head is 1 μm and contains a PSD (0.5 μm × 0.05 μm). Each spine neck is 0.2 μm wide and 0.5 μm long. Spines are spaced 1 μm apart on a dendritic segment (length, 10 μm; diameter, 1 μm). The photoactivated and adjacent spines are indicated with a solid and dashed square, respectively.
(E) Time course of fluorescence in the photoactivated spines (solid lines) and in their adjacent neighbors (dotted lines). Reaction parameters: *k*_on_ = 10/μM . sec; *k*_off _= 1/1,200 sec; *D_o_* = 10 μm/sec^2^. Initial concentrations (in μM): in the photoactivated PSD, photoactivated bound PSD-95 = 25, dark bound PSD-95 = 25, free binding sites = 5; in non-photoactivated PSDs, dark bound PSD-95 = 50, free binding sites = 5; ubiquitous unbound dark PSD-95 = 8.3 . 10^−4^. We varied the size of the photoactivated spine head and its PSD (relative to other spines): 0.5 (green), 1 (red), 2 (blue).
(F) Retention time as a function of spine size. Note that the retention times increased with increasing spine size, similar to the in vivo measurements (Figure 6D).
(3.8 MB TIF)

Figure S5 PSD Size and Spine Volume Are Correlated Comparison of PSD size and spine volume for pairs of spines. Each line corresponds to a pair of spines (black, positive slope, *n* = 32; gray, negative slope, *n* = 11, four animals). Spines with larger volumes, estimated from mCherry fluorescence, have larger PSDs, estimated from PSD-95-GFP fluorescence.
(1.1 MB TIF)

Protocol S1 Materials and Methods for Figure S1 and Supplemental Discussion (51 KB DOC)

## Abbreviations

L: layer
P: postnatal day
paGFP: photoactivatable GFP
PSD: postsynaptic density
