# Supplementary material for: Rapid Redistribution of Synaptic PSD-95 in the Neocortex In Vivo
Source: PLoS Biol. 2006 Nov 7;4(11):e370. doi: 10.1371/journal.pbio.0040370 (PMC1634879; doi:10.1371/journal.pbio.0040370)
Supplement: Supplementary file 1 [file pbio.0040370.sd001.doc]

# Protocol S1

**Materials and methods for Figure S1**

# Electrophysiology. To determine whether PSD-95-paGFP/mCherry expression modified synaptic circuitry, we mapped the synaptic inputs impinging on transfected and nearby (< 50 μm) non-transfected neurons using laser scanning photostimulation (LSPS; detailed methods in [1]) (Figure S1). Whole-cell recordings were obtained at room temperature in ACSF supplemented with 0.37 mM Nitroindolinyl (NI)-caged glutamate and 5 μM CPP (Sigma-RBI). The cells in a pair were recorded sequentially. There were no differences in their basic membrane properties (resting potential, input resistance, capacitance; *n* = 4 animals, 9 cell pairs, *P* > 0.09, Wilcoxon rank sum test). Focal photolysis of caged glutamate was accomplished with a 1 ms pulse of UV light (wavelength, 355 nm; DPSS Lasers, Inc.). Laser power was set to 20 mW at the specimen plane. The stimulus pattern for LSPS mapping consisted of 256 positions on a 16 × 16 grid, with 50 μm spacing. The grid overlaid L2/3, L4 and the top of L5. For each cell, we measured the mean amplitude of synaptic responses evoked in a window of 100 ms after the stimulus for each position in the grid. The mean amplitude of synaptic responses recorded in the transfected cells (plotted in Y) was indistinguishable from the mean amplitude of synaptic responses recorded in their non-transfected neighbors (plotted in X) (*P* > 0.5, Wilcoxon rank sum test).

**Supporting discussion**

The fluorescence images show that PSD-95 is highly concentrated in spines (Figure 1), where it associates with its ‘binders’ in the PSD (Figure S4A and S4B). PSD-95 unbinds with rate constant *k*_off_, and binds with rate constant *k*_on_. After unbinding, PSD-95 can escape the spine and diffuse along the dendrite to other spines.

**Short-time diffusion of PSD-95.** Here we consider the short-time diffusion of unbound PSD-95-paGFP before it has time to associate with a PSD. The low concentration of unbound PSD-95-paGFP in the dendrite allowed only a rough estimate of the free diffusion coefficient of PSD-95, *D*_o_. After photoactivating a 1 μm length of dendrite, the fluorescence signal equilibrated over a ~ 3 μm stretch of dendrite within 0.5 sec (our sampling interval), implying that *D*_o_ = x^2^ / 2T >> 1 μm^2^/s. This is significantly faster than diffusion coefficients for proteins that are anchored in the membrane (unpublished measurements) [2], implying that dendritic PSD-95-paGFP diffuses as a cytoplasmic species. *D*_o_ for PSD-95-paGFP is bounded from above by the diffusion coefficient of GFP in the cytoplasm, ~ 27 μm^2^/s [3]. Since PSD-95-paGFP is a large multi-domain protein, its diffusion coefficient is expected to be smaller than for GFP. Assuming that the diffusion coefficient scales as MW^1/3^ (MW paGFP = 27 kD; MW PSD-95-paGFP = 107 kD [4]) we expect *D*_o_ ~ 17 μm^2^/s.

**Long-time diffusion of PSD-95.** Here we consider the long-time diffusion of PSD-95-paGFP as it samples, and is trapped by, multiple immobile PSDs. Over long times (*t* > than *τ*_r_) photoactivated molecules (PSD-95-paGFP*, *P*) will spread along the dendrite with an effective diffusion coefficient, *D*_e_. The number of *P* at the *i*th synapse is given by (photoactivation at *t* = 0; x_0_ = 0):

$P\left(i,t\right)=P\left(i=0,t=0\right)\delta\left(4\pi D_{e}t\right)^{-1/2}\exp\left(-x_{i}^{2}/4D_{e}t\right)$ (S1)

*D*_e_ is the weighted sum of the diffusion coefficient of the bound species (immobile, *D*_b_ = 0) and the diffusing species [5]:

$$D_{e}=fD_{o}$$

where *f* is the fraction of free PSD-95. The concentration of PSD-95 at long times will decrease as *t^‑1/2^*.

The vast majority of PSD-95-GFP was in puncta, mostly in dendritic spines, with little diffuse fluorescence (Figure 1). This implies that *f* << 1. *f* can be quantified as the ratio $D_{e}/D_{o}$. *D_e_* can be measured from the spread of green fluorescence over time. The low signal levels precluded direct fitting of Equation S1. Instead we measured the integrated fluorescence in a 44-μm dendritic segment after photoactivating a single spine. After 90 minutes, 62 ± 18 % (mean ± standard deviation, *n* = 8) of the fluorescence remained in the field of view. From Equation S1 this implies *D*_e_ ≈ 0.05 μm^2^/s. The free fraction is then *f* = *D*_e_/*D*_o_ ~ 0.005 (range 0.01-0.001).

**Estimate of the time constants.** It is possible to derive order of magnitude estimates of time-constants. The diffusion time between synapses is given by their spacing, *δ* ~ 1 μm, and the relationship *τ*_D_ = *δ*^2^/2*D*, giving *τ*_D_ ~ 0.05 s.

Since *f* = *τ*_on_ / *τ*_off_, *τ*_on_ << *τ*_off_.

Here *τ*_on_ = 1 / (*k*_on_[R]) is the binding time of PSD-95 in the spine to the PSD. [R] is the concentration of unoccupied PSD-95 binders in the spine (Note that [R] is determined by the total concentration of the binders and their occupancy). Computer simulations over a wide range of parameter values (below) suggest that the scale of the retention time, *τ*_r_ (~ 1000 seconds), is set by *τ*_off_. Thus *τ*_on_ ≈ *f* *τ*_r_  ≈ 2 s.

For cytoplasmic paGFP *τ*_esc_ ≈ 0.5 s (Figure 3B). Since *τ*_esc_ = (*V*_sp_/ *D*_o_)*ℜ*_n_ [6,7], *τ*_esc_ for PSD‑95‑paGFP will be longer by the ratio of the free diffusion coefficients. The diffusion coefficient is lower for PSD‑95‑paGFP than paGFP (factor of ~ 2) and thus *τ*_esc_ ≈ 1.0 s. These estimates indicate that *τ*_on_ and *τ*_esc_ are similar in magnitude. *τ*_esc_ depends sensitively on spine geometry (Equation 1) (Figure S2).

To calculate the relationship between the retention time and *τ*_off_*, τ*_on_, and *τ*_esc_ we assume that *τ*_off_ *>> τ*_on_, *τ*_on_ ~ *τ*_esc ,_ *τ*_esc >>_ *τ*_diff_.

Diffusion between synapses along the dendrite is rapid. Depending on the relative magnitudes of *τ*_esc_ and *τ*_on_ an appreciable fraction of unbinding events are followed by rebinding, increasing the retention time. The average number of rebinding events before escape is given by *τ*_esc_ / *τ*_on_, giving Equation 1:

*τ*_r_ ≈ *τ*_off_ (1+ *τ*_esc_ / *τ*_on_ ).

**Synapse-specific capture and retention and the size of the PSD-95 cluster.** Synapse-specific differences in kinetic parameters will influence the sizes of PSD-95 clusters. This can be illustrated with a simple calculation. We assume that the total PSD-95 concentration is limiting and that PSD-95 molecules are bind independently. The number of PSD-95 molecules at the *i*th synapse is then given by:

d*N*_i_/d*t* = - *N*_i_/*τ*_r,i_ + *κ*_cap,i_

*κ*_cap,i_ is the rate of PSD-95 capture (Figure 4D).

*τ*_r,i_ is the retention time (Figure 5B).

At steady state:

*N*_i_= *κ*_cap,i_ *τ*_r,i_

Therefore the steady-state size of the PSD-95 cluster is expected to increase with increasing retention time and capture rate.

**Simulations.** We simulated diffusion along the dendrite using a Markov model (Figure S4B-S4D) and a realistic compartmental model (Figure S4E and S4F). The state diagram of the Markov model is shown in Figure S4B. For realistic ranges of parameter values (see above) we find that *τ*_esc_ modulates the retention time, *τ*_r_ (Figure S4C), consistent with Equation 1. *τ*_on_ plays a similar role (not shown). As expected, *τ*_r_ is proportional to *τ*_off_ (Figure S4D). Thus spine geometry (via *τ*_esc_), *k*_on_ and *k*_off_ all influence the retention time and competition between spines.

We also implemented a compartmental model in the Virtual Cell Modeling and Simulation Framework (University of Connecticut Health Center). We simulated a dendrite with dendritic spines in two dimensions (Figure S4E and S4F). The results are similar as from the Markov model. Retention time increased with increasing spine size (Figure S4F), consistent with the *in vivo* measurements (Figure 6), and as expected from Equation 1. Our simulations also revealed that the behavior of PSD-95 in vivo (Figures 1-6) imply that the concentration of PSD-95 binders exceeds the total concentrations of PSD-95. In this sense PSD-95 is limiting in the dendrite.

**Supporting references**

1. Shepherd GM, Pologruto TA, Svoboda K (2003) Circuit analysis of experience-dependent plasticity in the developing rat barrel cortex. Neuron 38: 277-289.

2. Groc L, Heine M, Cognet L, Brickley K, Stephenson FA, et al. (2004) Differential activity-dependent regulation of the lateral mobilities of AMPA and NMDA receptors. Nat Neurosci 7: 695-696.

3. Swaminathan R, Hoang CP, Verkman AS (1997) Photobleaching recovery and anisotropy decay of green fluorescent protein GFP-S65T in solution and cells: cytoplasmic viscosity probed by green fluorescent protein translational and rotational diffusion. Biophys J 72: 1900-1907.

4. Cho KO, Hunt CA, Kennedy MB (1992) The rat brain postsynaptic density fraction contains a homolog of the Drosophila discs-large tumor suppressor protein. Neuron 9: 929-942.

5. Sabatini BL, Maravall M, Svoboda K (2001) Ca^2+^ signaling in dendritic spines. Curr Opin Neurobiol 11: 349-356.

6. Svoboda K, Tank DW, Denk W (1996) Direct measurement of coupling between dendritic spines and shafts. Science 272: 716-719.

7. Bloodgood BL, Sabatini BL (2005) Neuronal activity regulates diffusion across the neck of dendritic spines. Science 310: 866-869.
